# Primary repair of colon injuries: clinical study of nonselective approach

**DOI:** 10.1186/1471-230X-10-141

**Published:** 2010-12-02

**Authors:** Ranko G Lazovic, Goran I Barisic, Zoran V Krivokapic

**Affiliations:** 1 Department of Abdominal Surgery, Clinical Center of Montenegro, Podgorica, Montenegro; 2 Department of Colorectal Surgery, First surgical clinic, Clinical centre of Serbia, Belgrade, Serbia

## Abstract

**Background:**

This study was designed to determine the role of primary repair and to investigate the possibility of expanding indications for primary repair of colon injuries using nonselective approach.

**Methods:**

Two groups of patients were analyzed. Retrospective (RS) group included 30 patients managed by primary repair or two stage surgical procedure according to criteria published by Stone (S/F) and Flint (Fl). In this group 18 patients were managed by primary repair. Prospective (PR) group included 33 patients with primary repair as a first choice procedure. In this group, primary repair was performed in 30 cases.

**Results:**

Groups were comparable regarding age, sex, and indexes of trauma severity. Time between injury and surgery was shorter in PR group, (1.3 vs. 3.1 hours). Stab wounds were more frequent in PR group (9:2), and iatrogenic lesions in RS group (6:2). Associated injuries were similar, as well as segmental distribution of colon injuries. S/F criteria and Flint grading were similar.

In RS group 15 primary repairs were successful, while in two cases relaparotomy and colostomy was performed due to anastomotic leakage. One patient died. In PR group, 25 primary repairs were successful, with 2 immediate and 3 postoperative (7-10 days) deaths, with no evidence of anastomotic leakage.

**Conclusions:**

Results of this study justify more liberal use of primary repair in early management of colon injuries.

**Trial registration:**

Current Controlled Trials ISRCTN94682396

## Background

Two surgical options have been described for treatment of colon injuries and each one has advantages and disadvantages; (a) those that include any type of fecal diversion, known as two stage management and (b) primary repair. Based on surgical experience in the Second World War, two stage procedure remained standard treatment for the next 35 years [1] in spite of insufficient scientific evidence. In late 1970 s, Stone and Fabian [2] performed first prospective randomized controlled trial using primary repair for colonic injuries in selected cases. They defined the so called "Stone and Fabian" exclusion criteria for primary repair of colonic injuries. These criteria have been questioned and modified by Flint and Vitale [3] in 1991, when more liberal attitude for primary repair emerged, based on substantial improvements of intensive care and data from non selected, randomized controlled trials. In 1999, Curran and Borzotta [1] reviewed 5400 cases of civilian colon injuries where more than a half of patients received primary repair. Exclusion criteria were re-evaluated again, leading to the conclusion that most previous reports were based on highly subjective surgical estimation of risk factors, so primary repair could be performed in consecutive number of patients without any exclusion criteria [4,5]. Prospective randomized trials performed in period 1995-96, compared results of primary repair with two stage procedure without using exclusion criteria [6,7]. They found that mortality and morbidity from abdominal sepsis were either similar or slightly lower in primary repair group, leading to the conclusion that only Penetrating Abdominal Trauma Index (PATI) > 25 is associated with slightly higher complication rate. In studies of nonselective randomized approach, Gonzales [7,8] concluded that all civilian injuries should be treated by primary repair. Numerous observational (Class 2) and retrospective (Class 3) studies [9-11], found better results of primary repair compared to two stage procedure, but there is a lack of randomized, class one studies. The problem of extensive colon injuries and the criteria for the method of repair remains controversial [12]. The aim of this study was to investigate the possibility of expanding indications for primary repair of colon injuries using nonselective approach.

## Methods

**Figure 1:**
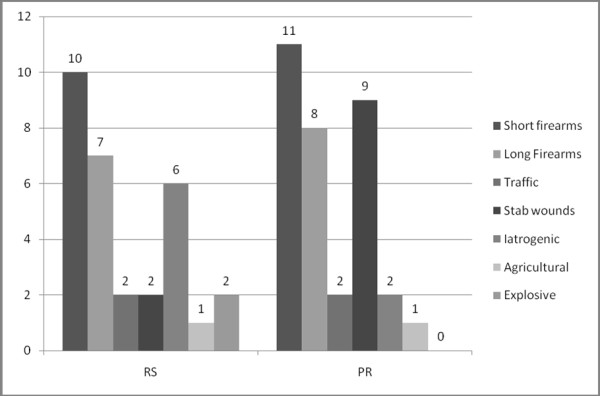
**Etiology of colon injury.** (PR-prospective group; RS-retrospective group)

This study was designed as retrospective and prospective evaluation of two stage procedure and primary repair in colon trauma management. Two groups of patients, one treated with selective approach and second treated with primary repair were analyzed in order to compare morbidity and mortality. The study was approved by the ethics committee of the Clinical Centre of Montenegro. Due to the severity of injuries and the need for urgent surgical treatment, it was not possible to seek informed consent from the patients, as a result, written informed consent was sought from a next of kin for participation in the trial. RS group included 30 patients (25 males and 5 females) with colon injury, treated in Clinical Centre of Montenegro, (CCM), Podgorica, in period 1995-2000. All patients in this group had war injuries and in all cases selective approach was used for the decision about the method of repair. PR group included 33 patients (29 males and 4 females) managed by same surgical team, in period 2000-2005. In this group, exclusion criteria were not used, with intention for primary repair in every case. The mean age in RS group was 36.8 years (SD 14.61, SE 2.66), and in PR group 41.3 years (SD 12.18; SE 2.17) with no statistical difference (T = 1.39; p > 0.05). Etiology of colon injuries varied between two groups (Figure 1). Iatrogenic injuries were more frequent in RS group (χ2 = 3.997), while stab wounds were more frequent in PR group (χ2 = 3.967), but overall distribution remained balanced (p > 0.05).

Isolated abdominal wounds were most common in both groups, 20 (66.8%) in RS group, and 19 (57.9%) in PR group followed by combined abdominal and chest injuries 6 (20%) in RS group and 7 (21.2%) in PR group. Overall distribution of concomitant injuries was similar in both groups (χ^2 ^= 1.047; p > 0.05).

In RS group main selection criteria were:

a. Trauma severity scores and indexes:

- Trauma score (TS) [13]

- Injury Severity Score (ISS) [14]

-Penetrating Abdominal Trauma Severity Index (PATI) [15]

b. Evaluation of general condition and abdominal findings at laparotomy:

- Stone and Fabian [2] criteria for primary repair of colon injury (SF)

- Flint grading [3] - contraindications for primary repair of colon injury (Fl)

Selection criteria were used for decision making regarding primary repair or diversion procedure in each case.

In period 2000-2005, based on encouraging experience from the RS group, all patients with colon injury were treated with primary repair, without any selection criteria except advanced peritonitis and multisegmental injuries of colon with impaired blood supply which are generally accepted as contraindications for primary repair.

The procedure of trauma management: after initial diagnostic and resuscitation procedures, patients were operated without any delay. In cases with associated multiple injuries treatment was conducted according to priority. The policy of primary repair included direct suture or resection with primary anastomosis. Antibiotic prophylaxis with 3^rd ^generation Cephalosporines and Metronidazole was standard part of the procedure.

Patients were discharged from hospital after restoring digestive function, and abdominal wound healing, usually 12^th ^to 14^th ^postoperative day. In all lethal cases autopsy was performed.

Trauma severity scores and indexes were calculated according to the methods described in the literature. Standardized statistical tests were used to determine group variables. For comparison between groups, T-frequency comparison test, Pearson's χ^2 ^test, Fisher's, and Test of Variances were applied.

## Results

The mean time between injury and admission to surgery (Latent time) in RS group was 3.1 hours (SD 3.41; SE 0.6) and in PR group 1.38 hours (SD 1.18; SE 0.24), revealing significant difference (T = 8.31; p < 0.01) in favor of PR group.

Trauma severity index showed no statistical difference between groups (p > 0.05) as shown in Table 1.

**Table 1:** Trauma Indexes

INDEX	RETROSPECTIVE(n = 30)	PROSPECTIVE(n = 33)	T	P
TS	15.55 (SD 16.70)	12.52 (SD 3.14)	.97	> .05

ISS	25.6 (SD 10.50)	23.3 (SD 8.30)	.10	> .05

PATI	23.4 (SD 12.70)	25.8 (SD 16.80)	.75	> .05

S/F	2.23 (SD 1.28)	2.15 (SD 1.40)	.03	> 0.5

FLINT	2.13 (SD 0.76)	1.97 (SD 0.70)	.84	> .05

(TS: Trauma Score; ISS: Injury Severity Score; PATI: Penetrating Abdominal Trauma Index; S/F: Stone/Fabian Criteria; F: Flint's grade)

There was statistical difference in PATI score between groups in category of three and four injuried abdominal organs (Table 2) in favour of PR group (T = 3.983 and 3.645). There was no statistical difference between RS and PR group in Stone/Fabian criteria. Flint grading was higher in PR group in category of four organs injuried (T = 3.124; p < 0.05). However, overall balance in indexes of local trauma remained similar in both groups (χ2 = 1.378, P > 0.05).

**Table 2:** Number of abdominal organ injured, PATI score, Stone-Fabian criteria and Flint's grade

No. of patients				PATI (+ SD) score		S/F criteria		FL grade	
**No Org**.	**RS**	**PR**	**Χ2**	**RS**	**PR**	**RS**	**PR**	**RS**	**PR**

1 organ	8	11	1.954	13.5(SD4.80)	12.1SD2.82)	1.44	1.21	1.88	1.5

2 organs	10	8	0.958	17.3(SD6.6)	18.0(SD2.1)	2.0	1.37	1.70	1.61

3 organs	7	4	2.297	29.1(SD7.10)	35.0(SD8.15)	3.00	3.25	2.43	2.50

4 organs	4	7	2.297	32.2(SD3.8)	38.2(SD10.6)	3.0	3.8	2.40	3.68

> 4 org.	1	3	0.87607	72.0(SD8.1)	61.3(SD6.34)	4.0	4.60	3.00	2.66

**TOTAL**	30	33	23.4(SD13.)	25.8(SD16.8)	2.23	2.15	2.13	2.15	

Distribution and severity of colon injuries was balanced between groups (RS vs. PR): ascending colon (8:6); transverse colon (5:6); and sigmoid colon (5:5).

Most frequent associated injuries (RS vs. PR) were: small intestine (13 : 9, χ2 = 1.83, p > 0.05); spleen (2:9 χ2 = 4.62 p < 0.05); kidney (5:7); liver and diaphragm (5:5); retroperitoneal hematoma (4:3) and stomach (4:2). The incidence of injury of duodenum, pancreas, urinary bladder, ureter, caval vein) ranged from 1to 2, and overall distribution in both groups remained balanced (T = 0.53, p > 0.05).

There was no difference between RS and PR group in number of contraindications for primary repair procedure (F = 1.924 p > 0.05). Primary repair was more frequent in PR group (F = 6.115, p < 0.05). In two cases, complications of primary repair in RS group needed conversion to two stage procedure, resulting with two deaths. In PR group there were no anastomotic complications necessitating relaparotomy, but number of deaths in the subgroup of primary repair was higher (χ2 = 1.145 p > 0.05) as shown in Table 3.

**Table 3:** Number of Stone/Fabian Criteria and results of One Stage Repair procedure

**No**.				I Repair				Success				Failure		
**No**.	**RS**	**PR**	**χ2**	**P**	**RS**	**PR**	**χ2**	**P**	**RS**	**PR**	**χ2**	**P**	**RS**	**PR**

0	3	4	.07	> .05	3	4	.07	> .05	3	4	.071	-	-	-

1	5	8	.05	> .05	4	8	1.28	> .05	4	8	1.213	-	-	-

2	10	8	.63	> .05	8	7	.26	> .05	6	6	.034	-	2*	1^†^

3	6	7	.01	> .05	2	6	1.88	> .05	1	6	3.508	-	1^†^	-

4	5	4	.26	> .05	1	3	.87	. > .05	1	1	.003	-	-	2^†^

5	1	2	.25	> .05	-	2	-	-	-	-	-	-	-	2^†^

TOTAL	30	33	F = 1.924	> .05	18	30	F = 6.115	< .01	15	25	F = 6.034	< .05	3	5

(* : Revisional complication; ^†^: Death)

The outcome of primary repair showed no statistical difference between two groups (χ2 = 1.034 p > 0.05). The same was found regarding two stage procedures (χ2 = 1.287 p > 0.05), as well as overall success rate of both procedures (χ2 = 0.22 P > 0.05). Significantly more common use of primary repair in PR group (n = 33; χ2 = 8.27; P < 0.05) resulted in higher succes rate (χ2 = 4.487 p < 0.05).

There were two anastomotic leakages in RS group necessitating relaparotomy. Bipolar colostomy (exteorisation) was performed in first and Hartmann procedure in second case. In PR group, two patients had to be reoperated due to complications of associated abdominal injuries (local abscess after pancreatic resection, and pararenal abscess), both with no signs of anastomotic leakage and with favorable outcome. Surgical procedures and results are shown in Table 4.

**Table 4:** Surgical procedures and results

PROCEDURE	No				SUCCESS				REVISION DEATH					
**ONE STAGE**	**Rs**	**Pr**	**χ2**	**P**	**Rs**	**Pr**	**χ 2**	**P**	**Rs**	**Pr**	**Rs**	**Pr**	**χ2**	**P**

**I Suture of colon**	14	22	2.56	> .05	12	19	1.947	> .05	2	-	-	3	-	-

**Right hemicolectomy**	4	4	. 02	> .05	3	3	.015	> .05			1	1	-	-

**Left flexure resection**	-	1	-	-	-	1	-	-	-	-	-	-	-	-

**Left hemicolectomy**	-	1	-	-	-	1	-	-	-	-	-	-	-	-

**Transverse colon resection**	-	1	-	-	-	-	-	-	-	-	-	1	-	-

**Primary suture of rectum**	-	1	3.88	< .05	-	1	2.866	-	-	-	-	-	.376	> .05

***Total. One Stage operation***	*18*	*30*	*8.27*	* < .05*	*15*	*25*	*4.487*	* < .05*	*2*	*-*	*1*	*5*	*.376*	* > .05*

**TWO STAGE**														

**Exteriorisation+colostomy**	2	1	-	-	2	-	-	-	-	-	-	1	-	-

**Prim. suture+colostomy**	4	-	4.58	< .05	4	-	7.29	< .05	-	-	-	-	-	-

**Hartmann's operation**	6	2	2.75	> .05	4	2	.964	> .05	1	-	1	-	-	-

***Total. One +Two Stage***	*30*	*33*	*-*	*-*	*25*	*27*	*.022*	* > .05*	*3*	*0*	*2*	*6*	*.025*	* > .05*

Postoperative mortality was higher in PR group (Fisher's test = 0.045, p < 0.05).

In this group, all deaths were caused by complications of associated injuries without signs of anastomotic leakage. One death in RS group was caused by anastomotic leakage after right hemicolectomy.

## Discussion

Concerning civil colon injuries, in 1993 Keighley [16] stated "... in experienced hands, using a very selective policy in low risk patients, repair of single laceration in two layers, after excising any irregular edges, appears to be optimal surgical approach" thus supporting the policy of primary repair of right colon and diversion procedure for left colon injuries. Nowdays, there is a definite trend toward increased use of primary repair in management of all penetrating colon injuries, independently of their localisation [17]. Numerous prospective randomized trials compared primary repair to diversion procedure, and demonstrated no significant difference in complication rates between groups [9,18]. Several recent reviews [19-21] analyzed the role of primary repair in treatment of colon injuries and pointed out that in conditions of similar intensity of general and local trauma, and similar intraoperative findings, primary repair had better results regarding complications, deaths and final outcome. Controversy remains only in cases of destructive colon injuries requiring resection, whether they should be treated with or without diversion procedure. According to AAST results of prospective multicenter trial [10,19] three risk factors for intraabdominal septic complications, independent from the method of repair were identified as: severe fecal contamination, transfusion of more than 4 blood units and single antibiotic prophylaxis. However, the concept of "severe fecal contamination" has not been clearly determined yet. The same author [10], comparing data from other reports, could not strongly support even these 3 criteria and stressed that there are only two main indications for performing two stage procedure: severe colon edema (whatever the cause) and questionable colon blood supply [19,20].

In this study, nonselective approach in favor of primary repair was used with very limited contraindications for primary repair. The mean latent time was shorter in PR group, which could be accounted for more favorable results. However, short latent time could also contribute for two early deaths in the PR group, because in case of longer delay they would not reach surgical service at all, due to severity of associated injuries.

Etiology of colon injuries was quite similar in both groups with differences in categories of iatrogenic injuries and stab wounds (Figure 1). In most cases, these injuries were similar in terms of severity of local and general trauma, so overall data were balanced. The intensity of general trauma and distribution to other body regions and organs were similar in both groups.

Severity abdominal trauma indexes (Table 2) were essentially similar in both groups, as well as number of injured organs. PATI score was slightly higher in PR group in the category of three and four organs injured (in both groups PATI > 25). Flint grading was higher in the category of four organs injured. Segmental distribution of colon injuries, as well as wound severity (Table 2 and 3), were equal. According to Stone/Fabian criteria, both groups were equal (Table 3).

Primary repair was performed in 60% of cases in the RS and in 90.9% in the PR group. Higher success rate of primary repair in the PR group (F = 6.034 p < 0.05), was mainly because S/F criteria was ≥3. This was probably the result of more liberal use of primary repair in higher categories of SF criteria, which is also supported by recent literature [21]. There was no significant difference regarding percentage of attempted and successful primary repairs in lower categories of S/F criteria between groups. The incidence of primary suture is the same in both groups (χ^2 ^2.56), but there are more resections with primary repair in PR group, thus achieving overall success in 25 of 30 attempted cases (F = 7.124 p < 0.05).

Two severe complications were registered in each group but in RS group they required conversion to two stage procedure. In RS group there was one more conversion procedure with lethal outcome. Complications in PR group were caused by associated injuries not requiring conversion procedure and ended favorably.

Mortality was higher in PR group (p = 0.045). There were 3 early postoperative deaths (two in category of one stage and one in category of two stage procedure) caused by severe injuries of other organs. There were also 3 late postoperative deaths, but none of them caused by colon injury. Analyzing unsuccessful cases together (complications and deaths), there was no statistical difference between two groups (χ^2^= 0.859 P > 0.05).

## Conclusions

According to our experience, we believe that the policy of primary repair of colon injuries can be applied more liberally in majority of patients with high success rate.

## Competing interests

The authors declare that they have no competing interests.

## Authors' contributions

RL consultant surgeon, conceived of the study, and participated in its design and coordination and helped to draft the manuscript

GB conceived of the study, and participated in its design and coordination and drafted the manuscript

ZK participated in the study design and helped to draft the manuscript

All authors read and approved the final manuscript.

## Pre-publication history

The pre-publication history for this paper can be accessed here:

http://www.biomedcentral.com/1471-230X/10/141/prepub
